# Supplementary material for: Engineering viral vectors for acoustically targeted gene delivery
Source: Nat Commun. 2024 Jun 10;15:4924. doi: 10.1038/s41467-024-48974-y (PMC11164914; doi:10.1038/s41467-024-48974-y)
Supplement: Supplementary file 1 — Supplementary Information [file 41467_2024_48974_MOESM1_ESM.pdf]

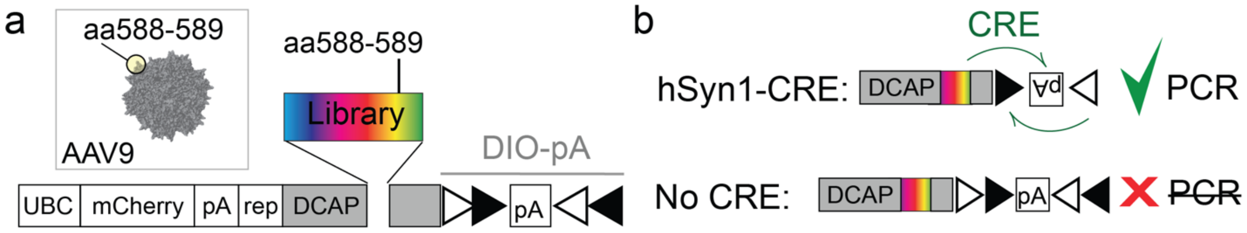

Supplementary Figure S1. Construction of the AAV library and CRE-dependent PCR. a, Randomized 21-basepair DNA fragment was inserted into the AAV9 capsid between amino acids 588 and 589, which resides at the exterior of an AAV capsid (inset). AAV capsid was produced within the AAV genome allowing for recovery of the capsid sequenced from transduced cells. The capsid coding sequence was followed by a polyA (pA) sequence flanked by a double-inverted floxed open reading frame (DIO). **b**, The DIO sequence can be recombined and inverted in the presence of Cre enzyme. That sequence inversion can then be detected using PCR. Therefore, the DNA from AAVs that transduced cells expressing Cre can be amplified using a PCR reaction. In our study, we used hSyn1-Cre mice which express Cre selectively in neurons, and thus, we selected for neuron-transducing AAVs.

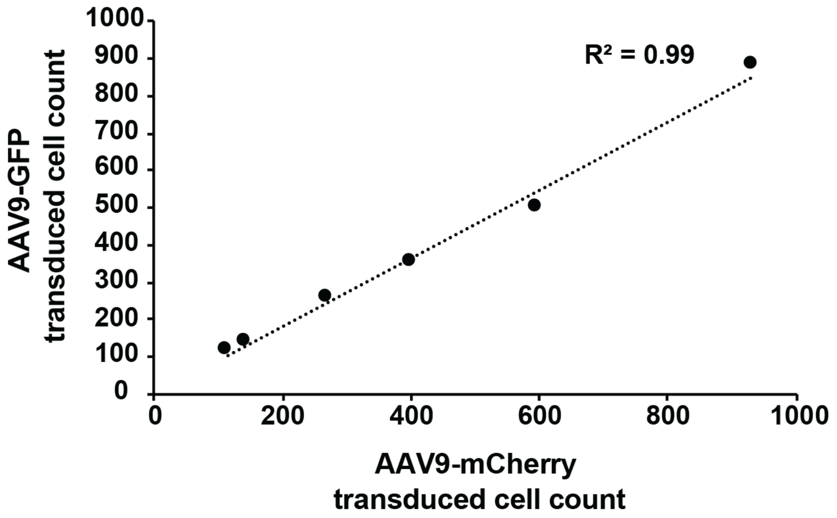

Supplementary Figure S2. AAV9 GFP + AAV9 mCherry. Transduced cell counts in the brain comparing AAV9 carrying GFP and mCherry are highly correlated and (Rsq=0.99). The AAVs were administered at 1E10 VGs per gram of body weight to each animal. The mean numbers of transduced cells are not significantly different (fold difference between AAV9-GFP and AAV9-mCherry: 1.07-fold, p=0.081 (ns), paired t-test, 6 sections tested from 2 mice).

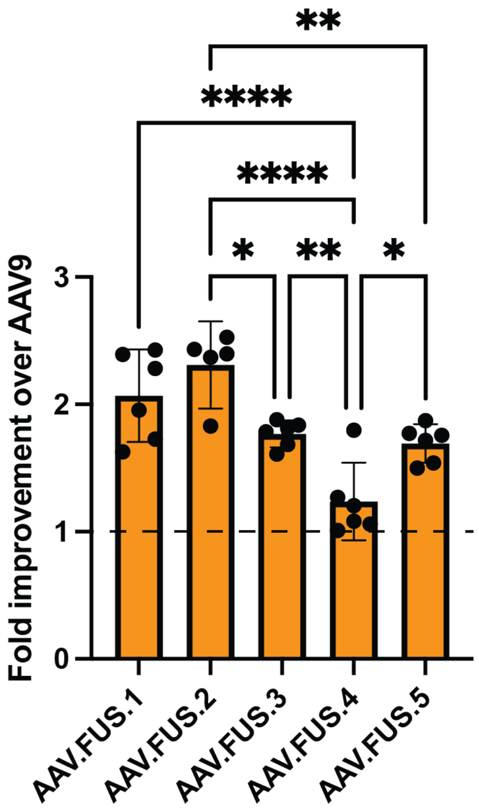

Supplementary Figure S3. Pairwise comparison of AAV.FUS candidates' transduction of the brain. Non-significant comparisons not shown for clarity**.** (**** = p<0.0001; *** = p<0.001; ** = p <0.01; * = p<0.05; F(4, 24) = 14.96, P<0.0001, One-way ANOVA with Tukey HSD post hoc test.). Non-significant pairwise comparisons not shown for clarity. Error bars are 95% CI. All 10 detailed p-values provided in the source data appendix. n=6 mice used for all serotypes except AAV.FUS.2 which used n=5 mice. IV injection dose, 10^{10} vg/g

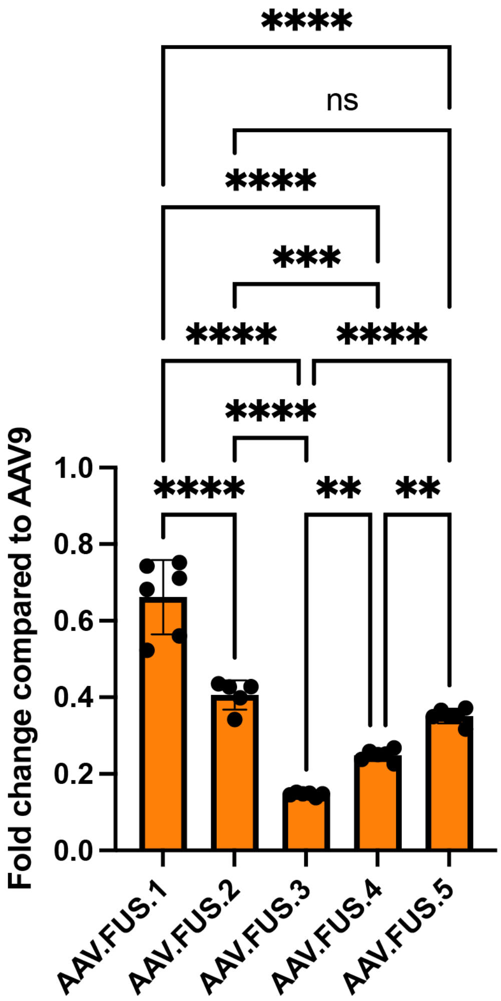

Supplementary Figure S4. Pairwise comparison of AAV.FUS candidates' transduction of the liver. Non-significant comparisons not shown for clarity. AAV.FUS.3 shows significantly reduced liver transduction compared to other AAV.FUS candidates. One-way ANOVA with Tukey HSD post-hoc test. $F(4, 24) = 96.69$. $P<0.0001$; All pairwise comparisons are below $p<0.0001$, except AAV.FUS.2 vs AAV.FUS.4 ($p=0.0001$), AAV.FUS.2 vs AAV.FUS.5 ($p=0.3524$), AAV.FUS.3 vs AAV.FUS.4 ($p=0.01$), and AAV.FUS.4 vs AAV.FUS.5 ($p=0.0099$)). (**** = $p<0.0001$; *** = $p<0.001$; ** = $p<0.01$; * = $p<0.05$, ns = non-significant). Error bars are 95% CI. Detailed p-values provided in the source data appendix. n=6 mice used for all serotypes except AAV.FUS.2 which used n=5 mice. IV injection dose, 10^{10} vg/g

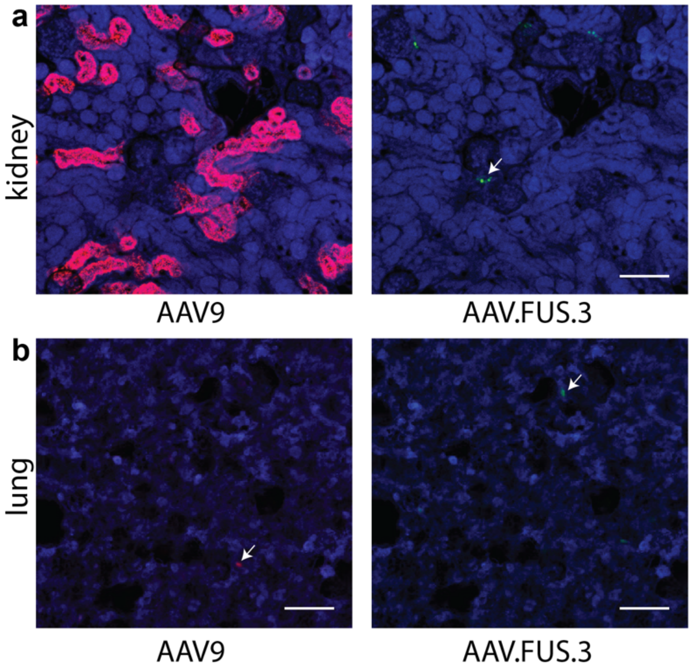

Supplementary Figure S5. Transduction of AAV9 and AAV.FUS.3 in peripheral tissues. We observed no substantial transduction observed in the **a)** kidneys and **b)** lungs, consistent with previous reports[21]**.** Representative images were obtained from mice co-injected with AAV9 (red, mCherry) and AAV.FUS.3 (green, GFP). IV injection dose, 10^{10} vg/g. Sections were imaged on a confocal microscope with 10x objective counterstained with a nuclear stain (DAPI, blue). Example positive cells designated with arrows. Scale bars are 100 microns.

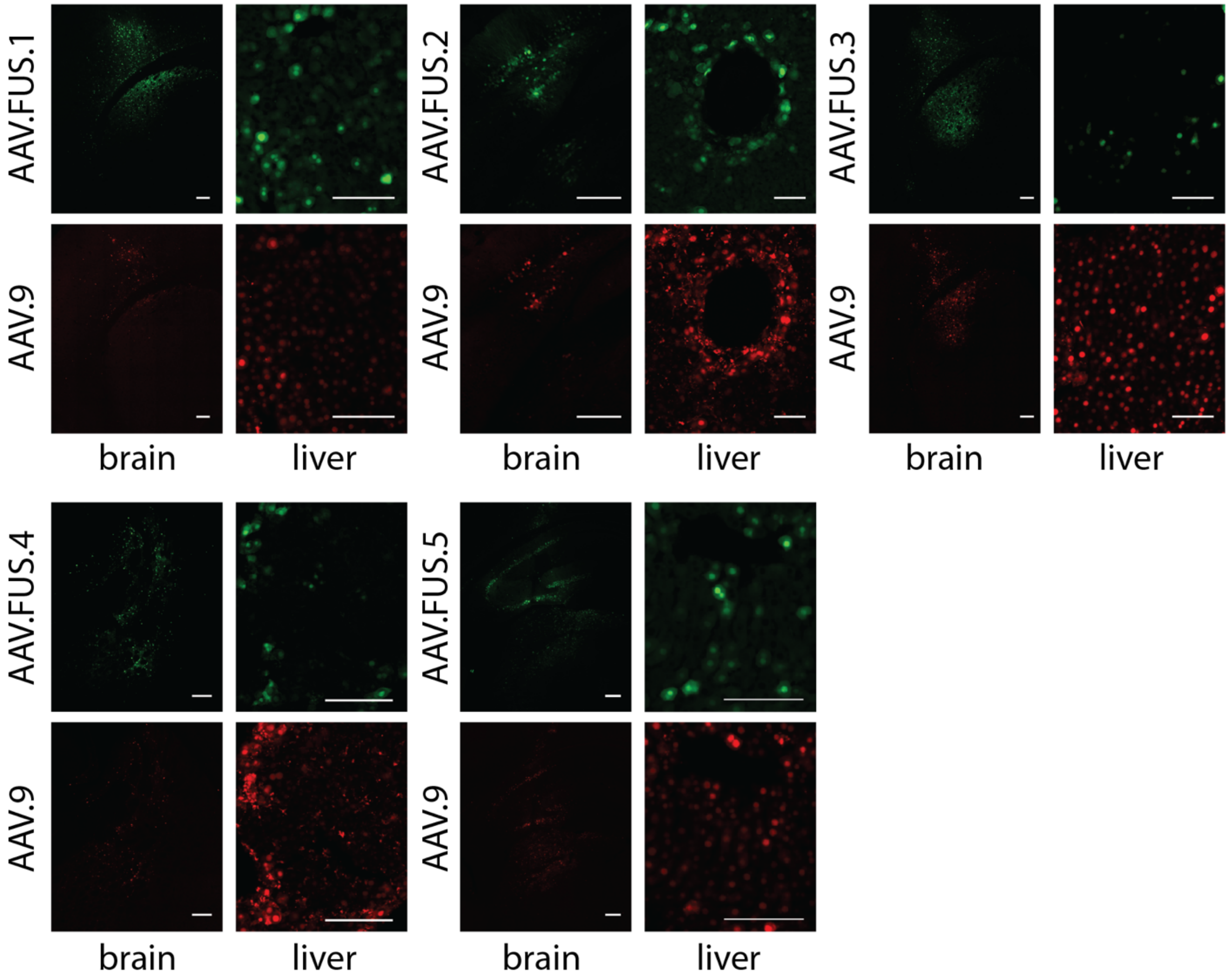

Supplementary Figure S6. Representative images of transduction in brain and liver for all AAV.FUS (green, EGFP) and corresponding co-injected AAV9 control (red, mCherry). IV injection dose, 10^{10} vg/g. Scale bars, 200 microns for the brain, 100 microns for the liver.

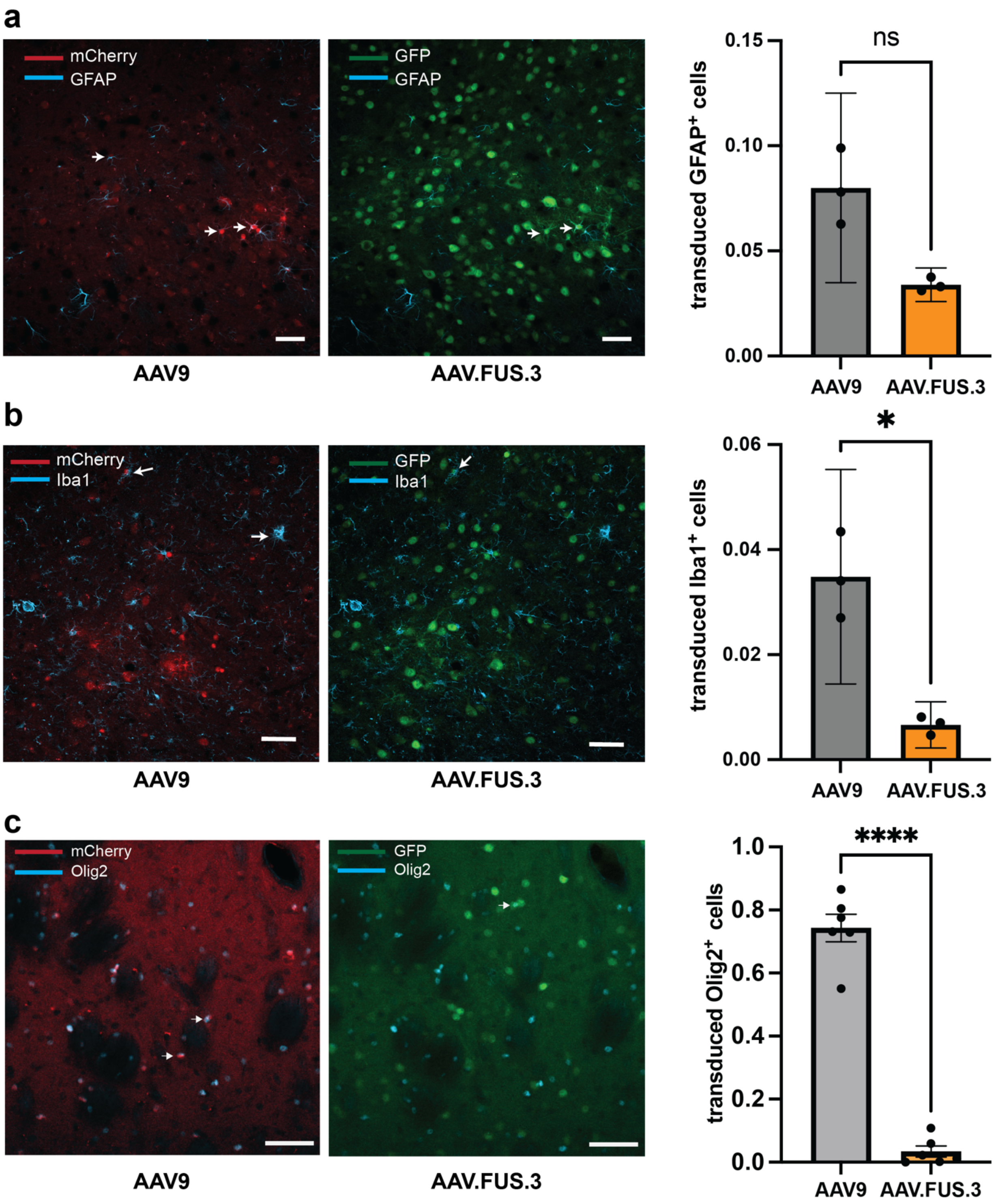

Supplementary Figure S7. Transduction of non-neuronal brain cells by AAV.FUS.3 and AAV9. We observed lower transduction of microglia/macrophages, astrocytes and oligodendrocytes in the brain by either AAV9 or AAV.FUS.3 as compared to neurons. Representative images were obtained from mice co-injected with AAV9 (red, mCherry) and AAV.FUS.3 (green, GFP**). a**) The transduction of astrocytes (GFAP^{+}) was comparable between the AAV9 and AAV.FUS.3, with 8% and 3.4% average transduction, respectively (n=3 mice,

p=0.0552, two-tailed paired t-test; t=4.076). **b**) Similarly, microglial/macrophagic transduction was also less efficient than neuronal transduction for both serotypes with AAV9 transducing 3.5% and AAV.FUS.3 transducing 0.7% of microglial cell, which was a statistically significant difference (n=3 mice, p=0.0174, two-tailed paired t-test, t=7.487). **c**) AAV.FUS.3 transduction of oligodendrocytes was significantly reduced relative to AAV9, with average transduction being 3.4% for AAV.FUS.3 and 74.3% for AAV9 (n=6 mice, p<0.0001, two-tailed paired t-test, t=12.32). Sections were imaged on a confocal microscope with 20x objective counterstained for glial cells (GFAP, blue), microglia/macrophages (Iba1, blue) and oligodendrocytes (Olig2, blue). IV injection dose, 10^{10} vg/g. Scale bars are 50 microns.

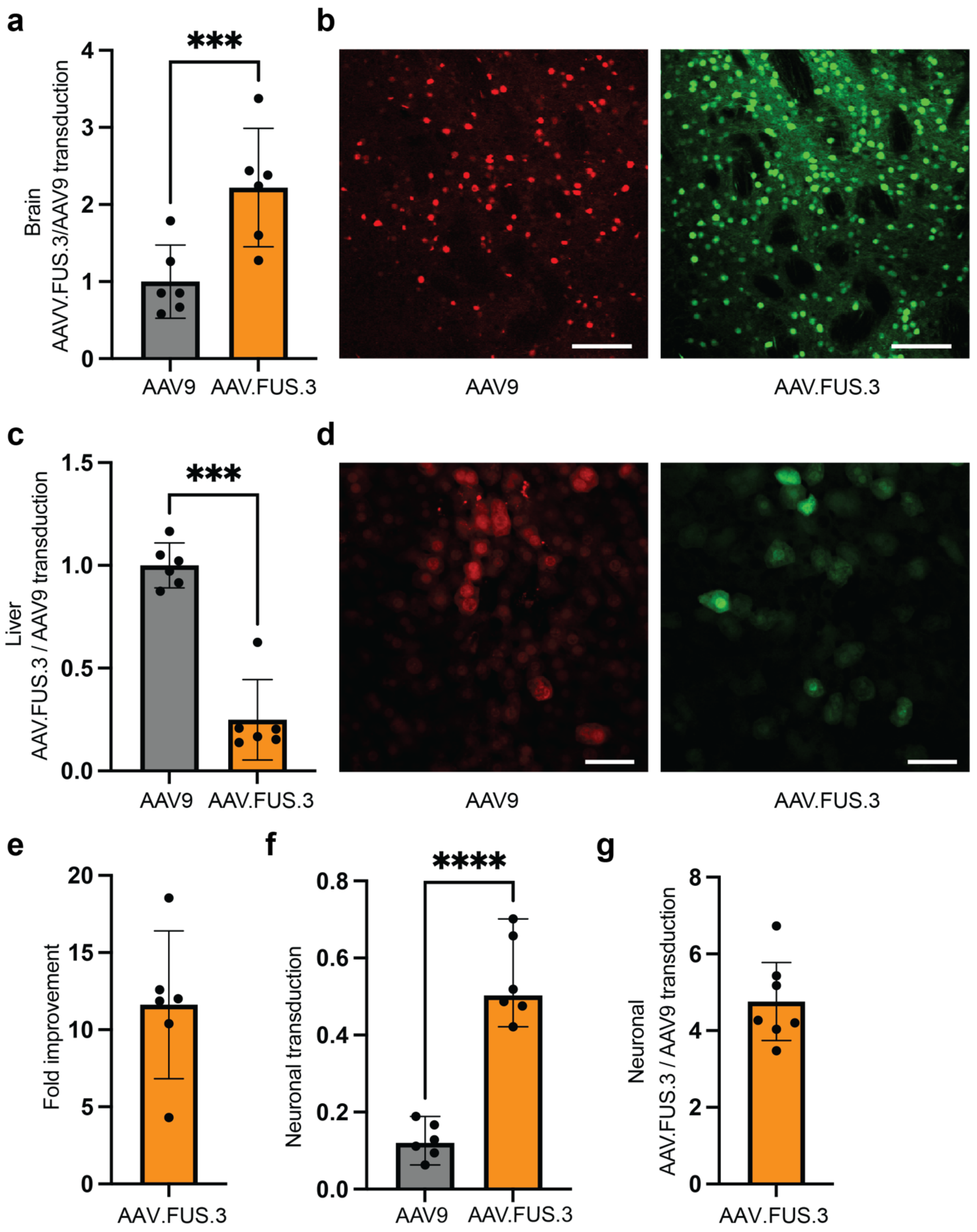

Supplementary Figure S8. AAV.FUS.3 delivered at $1x10^9$ vg/g of body weight shows reduced liver transduction and improved neuronal transduction efficiency. a, Representative images were obtained from mice co-injected with AAV9 and AAV.FUS.3 at $1x10^9$ viral genomes (vg) per gram of body weight. Sections were imaged on a confocal microscope

with 20x objective showing brain transduction by AAV9 (red) and AAV.FUS.3 (green). (n=6 mice, p=0.0004, two-tailed paired t-test; t=8.182). **b,** All mice showed improvement in transduction and expression over the co-injected AAV9 across various brain regions. **c,** Representative images from a confocal microscope with a 10x objective showing liver transduction by AAV9 (red) and AAV.FUS.3 (green) at the low dose (n=6 mice, p=0.0004, two-tailed paired t-test; t=8.53. **d,** The transduction of AAV.FUS.3 relative to AAV9 in the liver decreased significantly as the viral dose is reduced. **e**, We defined the fold-improvement in targeting efficiency as the ratio of brain transduction to the liver transduction efficiency using AAV9 as a baseline, showing 11.6-fold improvement in targeting efficiency at the dose of $1x10^9$ vg/g of body weight. **f**, Neuronal transduction at this tested dose was significantly higher for AAV.FUS.3 over AAV9 (n=6 mice, p<0.0001; two-tailed paired t-test; t=14.81). **g**, Overall, we observed 4.6-fold higher neuronal transduction by AAV.FUS.3 over AAV9. Scale bars are 100 microns in panel b and 50 microns in panel d. (**** = p<0.0001, *** = p<0.001; ** = p <0.01; * = p<0.05, ns = not significant).

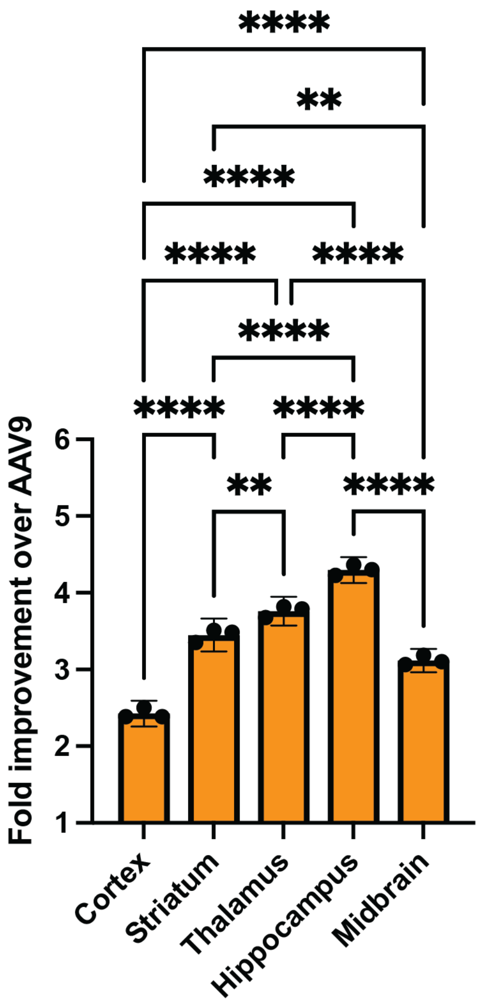

Supplementary Figure S9. Detailed pairwise comparisons for analysis of regional dependence of transduction efficiency for AAV.FUS.3. (**** = p<0.0001; *** = p<0.001; ** = p <0.01; * = p<0.05, ns = not significant; One-way ANOVA with Tukey HSD post-hoc test.). Detailed

p-values can be found in the source data appendix. n=3 mice used for all regions. IV injection dose, 10^{10} vg/g.

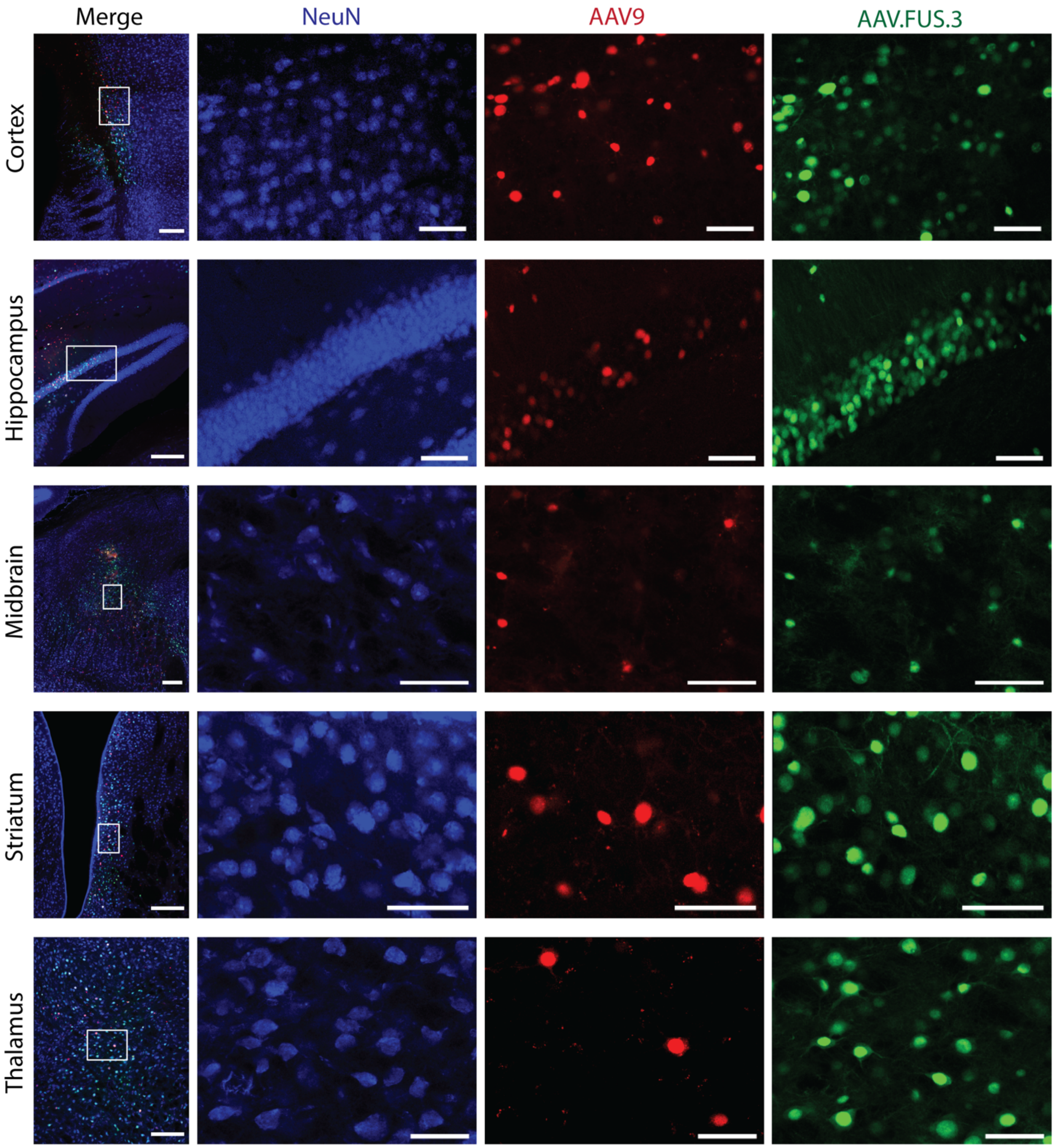

S10. Region-specific transduction of AAV9 (red) and AAV.FUS.3 (green) with a neuronal nuclear counterstain (NeuN) in C57BL/6J animals. Images were obtained at 20x magnification (left panels, scale bars 200 μm; 3 rightmost panels, scale bar 50 μm).

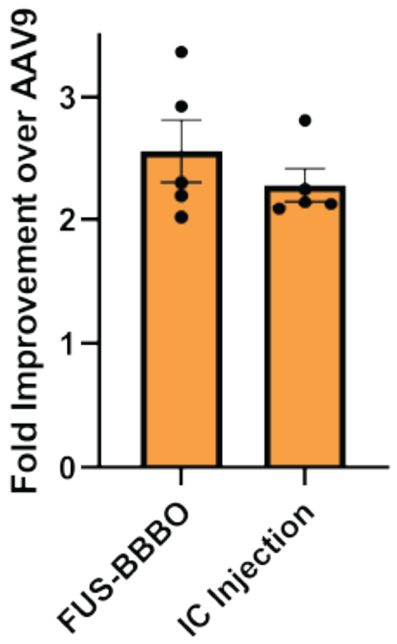

Supplementary Figure S11. Relative efficiency of neuronal transduction for AAV.FUS.3 and AAV9 upon intraparenchymal injection. The intraparenchymal injection dose was $4x10^8$ viral genomes into the CA1 of hippocampus. (n=5 mice per group, p=0.3694, two-tailed unpaired t-test; t=0.9689) while the IV injection dose for FUS-BBBO was 10^{10} vg/g.

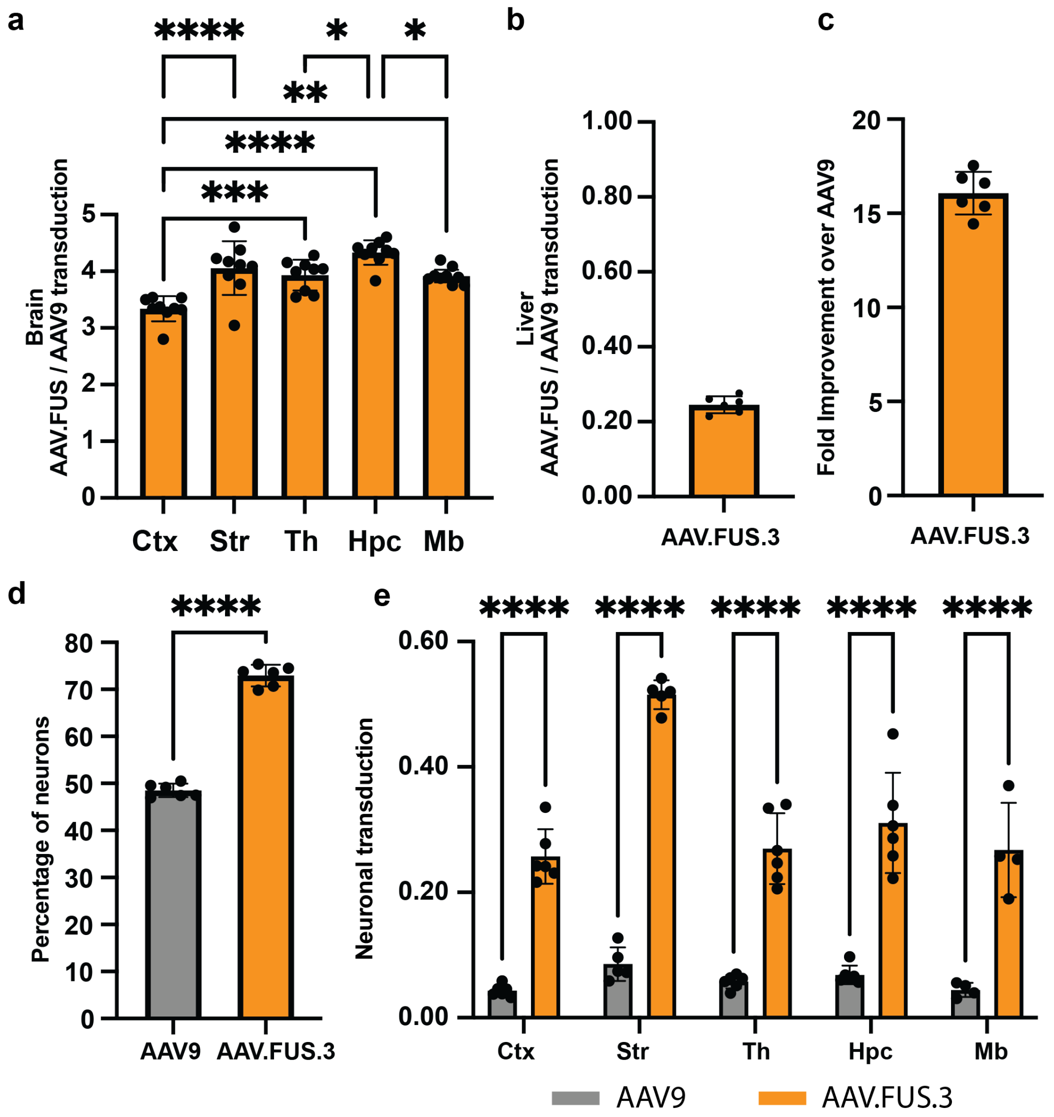

Supplementary Figure S12. AAV.FUS.3 shows similar transduction efficiency in BALB/cJ while maintaining neuronal transduction efficiency. a, Fold-improvement of the total number of transduced cells in the targeted brain areas by AAV.FUS.3 over AAV9 shows hippocampus (Hpc) is mostly highly transduced, and all the other tested regions (cortex (Ctx), Striatum (Str), Thalamus (Th), and Midbrain (Mb)) also showed improved transduction (F (4,40), 14.23, p<0.0001, one-way ANOVA). **b,** AAV.FUS.3 transduces the livers in BALB/cJ mice less efficiently than AAV9 (4.1±0.3-fold reduction). **c,** Improvement in brain-to-liver transduction ratio (n=6 mice)**. d,** Neuronal transduction is improved with AAV.FUS.3 over AAV9 (16.1±0.9-fold improvement, n=6 mice per group, p<0.0001, two-tailed paired t-test; t=21.48). **e,** Neuronal transduction efficiency for AAV9 (gray) and AAV.FUS.3 (orange). AAV.FUS.3 showed significant improvement over AAV9 transduction in all tested regions (two-way ANOVA with Sidak's test; F (1, 44) = 494.1; p<0.0001 for all tested regions). Scale bars are 200 microns in panel a and 100 microns in panel c. (**** = p<0.0001, *** = p<0.001; ** = p <0.01; * = p<0.05, ns = not significant). Error bars are 95% CI.

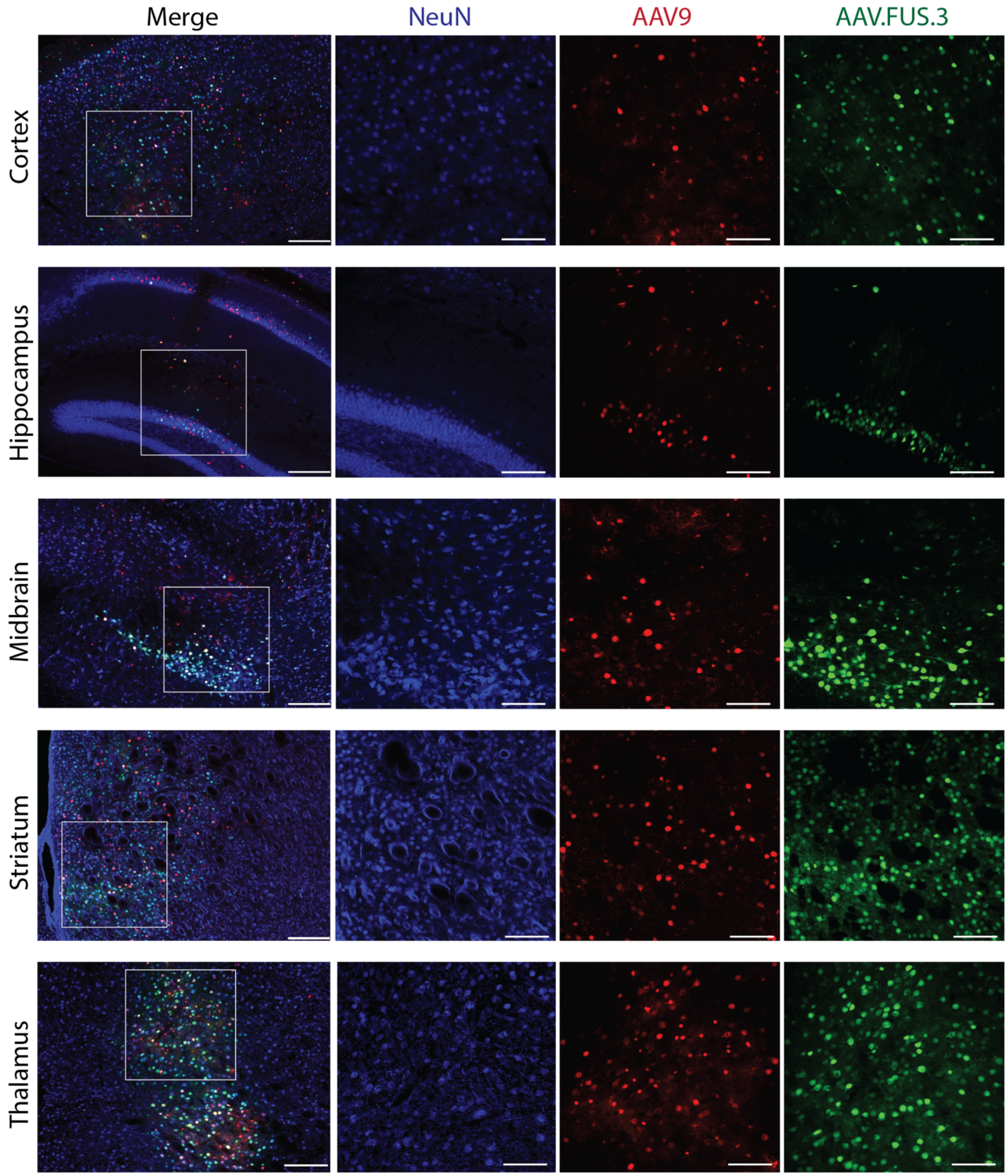

S13. Region-specific transduction of AAV9 (red) and AAV.FUS.3 (green) with a neuronal nuclear counterstain (NeuN) in balb/cJ mice. Images were obtained at 20x magnification (left panels, scale bars 200 μm; 3 rightmost panels, scale bar 50 μm).

Supplementary Table 1. Amino-acid sequences inserted into AAV9 capsid to obtain AAV.FUS.1-5 vectors.

Candidate	7-mer insert
AAV.FUS.1	AGNTSDR
AAV.FUS.2	ATDAYNK
AAV.FUS.3	WSEGGQP
AAV.FUS.4	SVGSADP
AAV.FUS.5	VRMEGEV

Supplementary Note 1. An open reading frame encoding the CAP protein of AAV.FUS.3 Highlighted region (yellow) indicates the site of insertion.

```
5′-ATGGCTGCCGATGGTTATCTTCCAGATTGGCTCGAGGACAACCTTAGTGAAGGAATTCGCG
AGTGGTGGGCTTTGAAACCTGGAGCCCCTCAACCCAAGGCAAATCAACAACATCAAGACAACGCTCGAGG
TCTTGTGCTTCCGGGTTACAAATACCTTGGACCCGGCAACGGACTCGACAAGGGGGAGCCGGTCAACGCA
GCAGACGCGGCGGCCCTCGAGCACGACAAGGCCTACGACCAGCAGCTCAAGGCCGGAGACAACCCGTACC
TCAAGTACAACCACGCCGACGCCGAGTTCCAGGAGCGGCTCAAAGAAGATACGTCTTTTGGGGGCAACCT
CGGGCGAGCAGTCTTCCAGGCCAAAAAGAGGCTTCTTGAACCTCTTGGTCTGGTTGAGGAAGCGGCTAAG
ACGGCTCCTGGAAAGAAGAGGCCTGTAGAGCAGTCTCCTCAGGAACCGGACTCCTCCGCGGGTATTGGCA
AATCGGGTGCACAGCCCGCTAAAAAGAGACTCAATTTCGGTCAGACTGGCGACACAGAGTCAGTCCCAGA
CCCTCAACCAATCGGAGAACCTCCCGCAGCCCCCTCAGGTGTGGGATCTCTTACAATGGCTTCAGGTGGT
GGCGCACCAGTGGCAGACAATAACGAAGGTGCCGATGGAGTGGGTAGTTCCTCGGGAAATTGGCATTGCG
ATTCCCAATGGCTGGGGGACAGAGTCATCACCACCAGCACCCGAACCTGGGCCCTGCCCACCTACAACAA
TCACCTCTACAAGCAAATCTCCAACAGCACATCTGGAGGATCTTCAAATGACAACGCCTACTTCGGCTAC
AGCACCCCCTGGGGGTATTTTGACTTCAACAGATTCCACTGCCACTTCTCACCACGTGACTGGCAGCGAC
TCATCAACAACAACTGGGGATTCCGGCCTAAGCGACTCAACTTCAAGCTCTTCAACATTCAGGTCAAAGA
GGTTACGGACAACAATGGAGTCAAGACCATCGCCAATAACCTTACCAGCACGGTCCAGGTCTTCACGGAC
TCAGACTATCAGCTCCCGTACGTGCTCGGGTCGGCTCACGAGGGCTGCCTCCCGCCGTTCCCAGCGGACG
TTTTCATGATTCCTCAGTACGGGTATCTGACGCTTAATGATGGAAGCCAGGCCGTGGGTCGTTCGTCCTT
TTACTGCCTGGAATATTTCCCGTCGCAAATGCTAAGAACGGGTAACAACTTCCAGTTCAGCTACGAGTTT
GAGAACGTACCTTTCCATAGCAGCTACGCTCACAGCCAAAGCCTGGACCGACTAATGAATCCACTCATCG
ACCAATACTTGTACTATCTCTCTAGAACTATTAACGGTTCTGGACAGAATCAACAAACGCTAAAATTCAG
TGTGGCCGGACCCAGCAACATGGCTGTCCAGGGAAGAAACTACATACCTGGACCCAGCTACCGACAACAA
CGTGTCTCAACCACTGTGACTCAAAACAACAACAGCGAATTTGCTTGGCCTGGAGCTTCTTCTTGGGCTC
TCAATGGACGTAATAGCTTGATGAATCCTGGACCTGCTATGGCCAGCCACAAAGAAGGAGAGGACCGTTT
CTTTCCTTTGTCTGGATCTTTAATTTTTGGCAAACAAGGAACTGGAAGAGACAACGTGGATGCGGACAAA
GTCATGATAACCAACGAAGAAGAAATTAAAACTACTAACCCGGTAGCAACGGAGTCCTATGGACAAGTGG
CCACAAACCACCAGAGTGCCCAATGGAGCGAGGGCGGCCAGCCCGCACAGGCGCAGACCGGTTGGGTTCA
AAACCAAGGAATACTTCCGGGTATGGTTTGGCAGGACAGAGATGTGTACCTGCAAGGACCCATTTGGGCC
```

AAAATTCCTCACACGGACGGCAACTTTCACCCTTCTCCGCTGATGGGAGGGTTTGGAATGAAGCACCCGC
CTCCTCAGATCCTCATCAAAAACACACCTGTACCTGCGGATCCTCCAACGGCCTTCAACAAGGACAAGCT
GAACTCTTTCATCACCCAGTATTCTACTGGCCAAGTCAGCGTGGAGATCGAGTGGGAGCTGCAGAAGGAA
AACAGCAAGCGCTGGAACCCGGAGATCCAGTACACTTCCAACTATTACAAGTCTAATAATGTTGAATTTG
CTGTTAATACTGAAGGTGTATATAGTGAACCCCGCCCCATTGGCACCAGATACCTGACTCGTAATCTGTA
A-3’

Supplementary Note 2: Alternative insertions for the other AAV.FUS candidates:

FUS.1 insert after 1764th nucleotide, 588th residue
GCGGGGAATACTAGTGATCGG

FUS.2 insert after 1764th nucleotide, 588th residue
GCCACCGACGCCTACAACAAG

FUS.3 insert after 1764th nucleotide, 588th residue
TGGAGCGAGGGCGGCCAGCCC

FUS.4 insert after 1764th nucleotide, 588th residue
AGCGTGGGCAGCGCCGACCCC

FUS.5 insert after 1764th nucleotide, 588th residue
GTGCGGATGGAGGGTGAGGTG
