# Supplementary material for: Engineering viral vectors for acoustically targeted gene delivery
Source: Nat Commun. 2024 Jun 10;15:4924. doi: 10.1038/s41467-024-48974-y (PMC11164914; doi:10.1038/s41467-024-48974-y)
Supplement: Supplementary file 2 — Reporting Summary [file 41467_2024_48974_MOESM2_ESM.pdf]

nature portfolio

Corresponding author(s): Mikhail G. Shapiro
Jerzy O. Szablowski

Last updated by author(s): Feb 21, 2024

Reporting Summary

Nature Portfolio wishes to improve the reproducibility of the work that we publish. This form provides structure for consistency and transparency in reporting. For further information on Nature Portfolio policies, see our Editorial Policies and the Editorial Policy Checklist.

Statistics

For all statistical analyses, confirm that the following items are present in the figure legend, table legend, main text, or Methods section.

n/a	Confirmed	
☐	☒	The exact sample size (*n*) for each experimental group/condition, given as a discrete number and unit of measurement
☐	☒	A statement on whether measurements were taken from distinct samples or whether the same sample was measured repeatedly
☐	☒	The statistical test(s) used AND whether they are one- or two-sided<br>*Only common tests should be described solely by name; describe more complex techniques in the Methods section.*
☐	☒	A description of all covariates tested
☐	☒	A description of any assumptions or corrections, such as tests of normality and adjustment for multiple comparisons
☐	☒	A full description of the statistical parameters including central tendency (e.g. means) or other basic estimates (e.g. regression coefficient) AND variation (e.g. standard deviation) or associated estimates of uncertainty (e.g. confidence intervals)
☐	☒	For null hypothesis testing, the test statistic (e.g. *F*, *t*, *r*) with confidence intervals, effect sizes, degrees of freedom and *P* value noted<br>*Give P values as exact values whenever suitable.*
☒	☐	For Bayesian analysis, information on the choice of priors and Markov chain Monte Carlo settings
☒	☐	For hierarchical and complex designs, identification of the appropriate level for tests and full reporting of outcomes
☒	☐	Estimates of effect sizes (e.g. Cohen's *d*, Pearson's *r*), indicating how they were calculated

Our web collection on statistics for biologists contains articles on many of the points above.

Software and code

Policy information about availability of computer code

Data collection	We used ThermoGuide v3846 (Image Guided Therapy) and Paravision v5.1 & v6 (Bruker) for MRI-guided focused ultrasound and MRI image acquisition. We used Morpheus software for focused ultrasound guidance on RK50 (FUS Instruments). We also used Zen Black v2.3 and Zen Blue v3.3 (Zeiss) for murine brain histological data collection.
Data analysis	We used Excel 2013 & 2019 (Microsoft), Prism v9 (Graphpad), lmageJ vl.SOa (NIH) and Zen Black v2.3 and Zen Blue v3.3 (Zeiss) for data and image analysis and plotting.<br>Illustrations were made in Illustrator CC (Adobe).

For manuscripts utilizing custom algorithms or software that are central to the research but not yet described in published literature, software must be made available to editors and reviewers. We strongly encourage code deposition in a community repository (e.g. GitHub). See the Nature Portfolio guidelines for submitting code & software for further information.

Data

Policy information about availability of data

All manuscripts must include a data availability statement. This statement should provide the following information, where applicable:

- Accession codes, unique identifiers, or web links for publicly available datasets
- A description of any restrictions on data availability
- For clinical datasets or third party data, please ensure that the statement adheres to our policy

The authors declare that all data supporting the findings of this study are available within the paper and its supplementary information. Raw data files are available from the corresponding author upon reasonable request.

Research involving human participants, their data, or biological material

Policy information about studies with human participants or human data. See also policy information about sex, gender (identity/presentation), and sexual orientation and race, ethnicity and racism.

Reporting on sex and gender	N/A
Reporting on race, ethnicity, or other socially relevant groupings	N/A
Population characteristics	N/A
Recruitment	N/A
Ethics oversight	N/A

Note that full information on the approval of the study protocol must also be provided in the manuscript.

Field-specific reporting

Please select the one below that is the best fit for your research. If you are not sure, read the appropriate sections before making your selection.

☒ Life sciences ☐ Behavioural & social sciences ☐ Ecological, evolutionary & environmental sciences

For a reference copy of the document with all sections, see nature.com/documents/nr-reporting-summary-flat.pdf

Life sciences study design

All studies must disclose on these points even when the disclosure is negative.

Sample size	Sample sizes were chosen on the basis of preliminary experiments so as to provide sufficient power for statistical comparison (where appropriate). Power analysis was not performed a priori.
Data exclusions	For validation experiment comparing AAV.FUS.2 mutant to the parent wild-type AAV, out of n = 6 mice (3 male and 3 female), n = 1 male deceased unexpectedly and prematurely, from which it was impossible to obtain any data for said experiment.
Replication	The number of independent biological replicates (n) is reported in the figure legends.
Randomization	All groups were randomized. Mice were randomized with regard to their administered AAV.FUS mutants in validation experiments.
Blinding	Data was blinded during histological analysis except for one data set (Fig. S7) that was not anonymized due to file-sharing error.

Reporting for specific materials, systems and methods

We require information from authors about some types of materials, experimental systems and methods used in many studies. Here, indicate whether each material, system or method listed is relevant to your study. If you are not sure if a list item applies to your research, read the appropriate section before selecting a response.

Materials & experimental systems

n/a	Involved in the study
☐	☒ Antibodies
☒	☐ Eukaryotic cell lines
☒	☐ Palaeontology and archaeology
☐	☒ Animals and other organisms
☒	☐ Clinical data
☒	☐ Dual use research of concern
☒	☐ Plants

Methods

n/a	Involved in the study
☒	☐ ChIP-seq
☒	☐ Flow cytometry
☐	☒ MRI-based neuroimaging

Antibodies

Antibodies used	All antibodies are commercially purchased from Novus Biological, Littleton, CO, and included: anti-RBFOX3/NeuN (1B7) Alexa Fluor 405-conjugated antibody (NBP1-92693AF405), anti-GFAP (5C10) Alexa Fluor 405-conjugated antibody (NBP1-05197AF405) and anti-AIF-1/Iba1 (rAIF1/1909) Alexa Fluor 405-conjugated antibody (NBP1-75760AF405).
Validation	All antibodies were validated by the manufacturer for immunohistochemistry applications.

Animals and other research organisms

Policy information about studies involving animals; ARRIVE guidelines recommended for reporting animal research, and Sex and Gender in Research

Laboratory animals	C57BL/6J, Balb/cJ and B6.Cg-Tg(Synl-cre)671JxmJ (3966) mice were obtained from JAX laboratories, with ages 10-18 weeks at the start of experiments. Animals were housed in 12-hour light/dark cycles and were provided water and food ad libitum. All experiments were conducted under a protocol approved by the Institutional Animal Care and Use Committees of the California Institute of Technology and of Rice University.
Wild animals	The study did not involve wild animals.
Reporting on sex	The groups used in all figures were 50% male and 50% female whenever possible (i.e. for even number of mice) except for supplementary figures.
Field-collected samples	The study did not involve samples collected from the field.
Ethics oversight	All animal procedures performed in this study were approved by California Institute of Technology and Rice University Institutional Animal Care and Use Committees (IACUC).

Note that full information on the approval of the study protocol must also be provided in the manuscript.

Plants

Seed stocks	N/A
Novel plant genotypes	N/A
Authentication	N/A

Magnetic resonance imaging

Experimental design

Design type	Static image
Design specifications	Single image
Behavioral performance measures	No behavioural performance was measured.

Acquisition

Imaging type(s)	FLASH
Field strength	7T
Sequence & imaging parameters	Flash, 3D, coronal: 3.9 ms TE, 15ms TR, 15 deg. flip angle; Voxel size: 200x200x351 micrometres; matrix: 130x130x114<br>Flash, 2D, axial: 3ms TE, lO0ms TR, 30 deg flip angle, voxel: 80x80x350 micrometres; matrix 225x225x8
Area of acquisition	Whole brains were scanned.

Diffusion MRI ☐ Used ☒ Not used

Preprocessing

Preprocessing software	No preprocessing was used.
Normalization	No normalization was used.
Normalization template	No template was used
Noise and artifact removal	No noise removal or artifact removal were used
Volume censoring	No volume censoring was used.

Statistical modeling & inference

Model type and settings	No statistical modeling was used.
Effect(s) tested	No statistical effects were tested.

Specify type of analysis: ☒ Whole brain ☐ ROI-based ☐ Both

Statistic type for inference<br>(See Eklund et al. 2016)	None used.
Correction	None needed.

Models & analysis

n/a	Involved in the study
☒	☐ Functional and/or effective connectivity
☒	☐ Graph analysis
☒	☐ Multivariate modeling or predictive analysis
